# Neither in vivo MRI nor behavioural assessment indicate therapeutic efficacy for a novel 5HT_1A _agonist in rat models of ischaemic stroke

**DOI:** 10.1186/1471-2202-10-82

**Published:** 2009-07-16

**Authors:** Maria Ashioti, John S Beech, Andrew S Lowe, Michel Bernanos, Andrew McCreary, Michel M Modo, Steve CR Williams

**Affiliations:** 1 Neuroimaging Research Group, Clinical Neuroscience – PO42, Institute of Psychiatry, Kings College London, De Crespigny Park, Denmark Hill, London SE5 8AF, UK; 2 Department of Medicine, University of Cambridge, Addenbrooke's Hospital, Hills Road, Cambridge, UK; 3 Solvay Pharmaceuticals, C.J. van Houtenlaan 36, 1381 CP Weesp, the Netherlands

## Abstract

**Background:**

5HT_1A _agonists have previously been shown to promote recovery in animal models of stroke using ex vivo outcome measures which have raised the hopes for a potential clinical implementation. The purpose of this study was to evaluate the potential neuroprotective properties of a novel 5HT_1A _agonist DU123015 in 2 different models of transient focal ischaemic stroke of varying severities using both in vivo neuroimaging and behavioural techniques as primary outcome measures. For these studies, the NMDA receptor antagonist MK-801 was also utilized as a positive control to further assess the effectiveness of the stroke models and techniques used.

**Results:**

In contrast to MK-801, no significant therapeutic effect of DU123015 on lesion volume in either the distal MCAo or intraluminal thread model of stroke was found. MK-801 significantly reduced lesion volume in both models; the mild distal MCAo condition (60 min ischaemia) and the intraluminal thread model, although it had no significant impact upon the lesion size in the severe distal MCAo condition (120 min ischaemia). These therapeutic effects on lesion size were mirrored on a behavioural test for sensory neglect and neurological deficit score in the intraluminal thread model.

**Conclusion:**

This study highlights the need for a thorough experimental design to test novel neuroprotective compounds in experimental stroke investigations incorporating: a positive reference compound, different models of focal ischaemia, varying the duration of ischaemia, and objective in vivo assessments within a single study. This procedure will help us to minimise the translation of less efficacious compounds.

## Background

Although the neurological consequences of stroke damage remain a leading cause of adult disability in industrialised countries [1], little progress has been achieved in translating promising experimental therapies into clinical practice. This lack of translational success has been thoroughly debated in recent years [2]. The main focus of drug development for stroke has been the investigation of neuroprotective compounds capable of protecting potentially salvageable neurons from ischaemic cell death. These potential neuroprotective compounds have, in the main, been successful in pre-clinical studies, but all bar an exceptional few have failed during early clinical trials, this being mostly due to lack of efficacy and potential adverse effects [3]. However, with an aging population and an increasing socioeconomic burden of stroke the persistent quest for potential neuroprotective agents is an absolute necessity [4].

One of the neuroprotective targets currently under investigation is the 5-hydroxytryptamine (5-HT) system, which includes 5HT_1A _receptor agonists. These compounds have been demonstrated to stimulate postsynaptic 5HT_1A _receptors located on the hippocampus and neocortex which are both vulnerable areas following ischaemia [5,6]. The activation of these receptors has been hypothesised to offset the excitotoxic properties of glutamate [7]. This is attributable to the hyperpolarisation of neuronal cells due to the activation of inwardly rectifying potassium channels [5]. 5HT_1A _agonists (e.g. Repinotan) have therefore been evaluated in models of ischaemic injury and have demonstrated substantial neuroprotective properties in both *in vitro *[8] and *in vivo *pre-clinical assessments [9-12]. However, these investigations have not included translatable in vivo measures of lesion severity, such as magnetic resonance imaging (MRI), or a direct comparison of efficacy in multiple models of cerebral ischaemia.

We here present a detailed *in vivo *investigation of the potential neuroprotective properties of a novel 5HT_1A _agonist, DU123015, for the treatment of transient focal cerebral ischaemia. For this, 2 animal models of ischaemia with varying occlusion times, different rodent strains, structural and behavioural outcome measures, and acute and sub-acute time-points are compared for the 5HT_1A _agonist in addition to a positive control compound (MK-801) that is known to be neuroprotective.

## Methods

All procedures were performed in accordance with the UK Animals (Scientific Procedures) Act of 1986 and the Ethical Review Panel of Queen Mary, University of London. All animals were acclimatised to their housing conditions for at least a week prior to the commencement of the study. Food and water were available *ad libitum*.

### Middle Cerebral Artery occlusion (MCAo)

#### Distal MCAo model

The surgical procedure was performed as described by Buchan et al. (1992). Briefly, adult male spontaneously hypertensive rats (SHR's, 200–250 g, Charles River, UK) were anaesthetised with 2% isofluorane in a mixture of 30% O_2 _and 70% N_2_O. Body temperature was monitored and maintained at 37°C using a heating blanket and rectal probe. O_2 _saturation was constantly above 95% saturation during the surgical procedure and heart rate remained within the correct margins of 380–420 bpm. The rat was positioned onto its left flank. An incision was then made between the right external auditory canal and the cantus of the eye. A burr hole was then drilled into the skull at the position where the zygomatic arch meets the squamozal bone to expose the right MCA. The dura was cut and retracted to expose the MCA which was then occluded with a number 1 microclip (Codman, Boston, Mass) placed proximally to the point where the MCA crosses the supracerbral vein in the rhinnal fissure. Immediately following the occlusion, the right CCA was occluded in order to reduce anterograde arterial perfusion [13]. The animals then regained consciousness during MCA occlusion before being reanaesthetized for reperfusion. All animals were monitored and weighed daily and a standardized post-operative care regime was implemented [14]. Animals were fed a moistened diet of breeding pellets and baby food (Heinz, UK) to facilitate food consumption and avoid weight loss. If weight loss did occur, animals were given a 5 ml Duphalyte and saline (50:50) injection i.p. daily for physiological support and to facilitate weight gain.

#### Intra-luminal thread model of MCAo

Male Sprague Dawley rats (280–360 g, Charles River, UK) were anaesthetised with 2% isoflurane in a mixture of 30% O_2 _and 70% N_2_O. Body temperature, O_2 _saturation and heart rate were all monitored as described above. The animals were then subjected to a 90 minutes MCAo using an adapted version of the method originally described by Koizumi et al. (1986) [15]. Briefly, an incision was made through the midline to expose the right common carotid artery. A 4-0 monofilament nylon suture with a silicone rubber coated 0.31 mm diameter tip was placed into the internal carotid artery via an external carotid arteriotomy and advanced approximately 21 mm from its original position to obstruct the blood flow to the MCA. The animals were allowed to fully recover from anaesthesia during the MCAo. Following 90 minutes of MCA occlusion, the animals were re-anaesthetized and the filament was retracted completely to allow reperfusion of the MCA.

### Neurological Score

A previously described battery of neurological tests comprising the grasping reflex (ability to grasp with both hands simultaneously), placing reaction, visual placing, righting reflex, tilted cage top test, horizontal bar test, spontaneous motility, circling and the tail lift test were conducted daily [14]. Each test was awarded either a zero for successful completion or a one indicating a neurological deficit. Therefore, the higher the overall score, the worse the neurological impairment. A non-parametric Kruskall-Wallis test was used for statistical analysis.

### Drug Preparation and administration

Animals were selected randomly from the cage and group assignment was alternated to yield the same number of animals in each group for any given day of surgery.

#### DU123015

DU123015 (European patent number: EP 0 650 964 A1) has a plasma half life of 0.7 – 1 hr. DU123015 is a selective 5-HT_1A _receptor agonist (pKi = 9.2) and has at least a 100 fold selectivity over other targets, e.g. 5-HT_1D _(pKi = 7.2). Receptor binding studies indicate that no binding (pKi < 6) was observed at other GPCRs, transporters or ion-channel targets. The putative nature of an interaction with the 5-HT_1A _receptor demonstrated that DU123015 was a full 5-HT_1A _agonist with a pEC50 of 8.0. Induction of the 5-HT_1A _behavioural syndrome was observed at 0.1 mg/kg i.v. EEG recordings demonstrating that total energy was reduced following DU123015 administration illustrating the compounds ability to penetrate the blood brain barrier and have an effect on global cortical brain function [See additional file 1].

A stock solution of DU123015 (Solvay Pharmaceuticals, Netherlands), was prepared daily at 4.4 mg/ml using sterile water by an experimenter that was not involved in the administration of the compound. For i.v. administration, an infusate of 4.4 μg/ml solution was prepared from the stock solution with 5% glucosaline. To evaluate the neuroprotective properties of DU123015, it was firstly administered as an intravenous bolus [35.2 μg/kg] immediately after the start of MCA occlusion. Following this bolus, DU123015 was continuously infused intravenously (i.v.) at a concentration of 8.8 μg/kg/hr i.v. for 4 hours in fully conscious rats at a rate of 0.5 ml/hour. This dose and route of administration is comparable to other agonists with a similar affinity and potency on the 5HT_1A _receptor [12,16,17]. The femoral vein was used for i.v. drug infusion using polyethylene tubing (ID 0.58 mm and OD 0.96 mm) which was inserted prior to MCA occlusion. A long catheter was used which was buried under the skin and exited at the back of the neck. This was attached to a swivel arrangement to permit continuous i.v. infusion of the drug to the conscious rat via an infusion pump. Control animals received an intravenous bolus loading dose of 5% glucosaline (Baxter, UK) at occlusion followed by a 4 hour continuous infusion of 5% glucosaline at the same infusion rate as DU123015 (i.e. 0.5 ml/hr).

#### MK-801

Dizocilpine (+)-MK-801 maleate salt (Sigma-Aldrich, UK) was dissolved daily in saline and administered intra-peritoneally (i.p.) as a bolus at a concentration of 1.5 mg/kg at occlusion. An i.p. 0.9% saline bolus injection served as a control.

### Magnetic Resonance Imaging

#### Acquisition

Magnetic Resonance Imaging was performed on a 4.7T Varian horizontal bore NMR spectrometer. A quadrature birdcage coil with a 63 mm internal diameter (Varian, USA) was used for RF transmission and reception. T2-weighted images (TE = 60 ms; TR = 2800 ms) were acquired for each animal at each time point [See additional file 2]. Total scanning time was 1 hour. The images were collected with a data matrix size of 192 × 192 over a field of view of 3.5 × 3.5 cm, yielding an in plane resolution of 182 μm with 40 contiguous 450 μm thick slices and six signal averages per phase encoding step.

Induction of anaesthesia consisted of 4% isofluorane in a mixture of 30% O_2 _and 70% N_2_O for approximately 5 minutes. Isofluorane concentration was then reduced to 1.5% and maintained at this concentration for the duration of MR image acquisition. Both respiration and heart rate were monitored during the scanning process as well as blood oxygen saturation using a pulse oximeter. Body temperature was monitored and maintained at 37°C with a heated blanket and feedback rectal temperature probe.

#### Post-processing of MRI

Absolute lesion and brain volumes were measured by converting voxel values into mm^3^. To account for individual differences in brain size, total brain volume was measured to determine % lesion over the entire brain. Lesion volumes were obtained using a semi-automated threshold process whereby the mean signal intensity in the cortex of the unaffected contralateral hemisphere was considered as a reference value to determine infarcted tissue [18]. The lesion was then delineated further using a semi-automated contouring system to discard hyperintensities emanating from the cerebral ventricles. To establish neuroprotection in relation to the control group, the mean volume of the control group was set at 100% to calculate the degree of infarct reduction in the respective treatment group. A GLM repeated measures ANOVA was then performed to detect any overall differences between the groups. A one way ANOVA was then also performed in order to identify differences between the groups at each time-point.

### Inclusion/Exclusion criteria

#### Distal MCAo

Two different criteria were applied for the exclusion of animals with a lesion using this method of MCAo; 1. Animals with no apparent lesion observed on the MRI scan at day 1 as a result of the clip were excluded from the study; 2. The remaining animals were then further screened using the extreme studentized deviate (ESD) method (Graphpad software Inc.) of detecting an outlier in order to remove unrepresentative animals

#### Intraluminal thread

Additionally to the 2 exclusion criteria for distal MCAO, animals that presented with a sub-arachnoid haemorrhage as observed by MRI on day 1 were also excluded from further analysis.

The application of exclusion criteria was applied whilst the experimenter remained blinded to treatment allocation. An overview of animals that were excluded in each condition are summarised in Table 1.

**Table 1:** Summary of the included and excluded animals for each condition.

**Model**	**Inclusion/Exclusion**	**DU123015**		**MK-801**	
		Drug	Control	Drug	Control
**Experiment 1** 120 minutes	Enrolled	8	10	9	7
	No Lesion	1	2	3	2
	Outlier	1	1	0	0
	***Included***	***6***	***7***	***6***	***5***
**Experiment 2** 60 minutes	Enrolled	10	18	13	12
	No Lesion	2	7	4	4
	Outlier	0	0	0	0
	***Included***	***8***	***11***	***9***	***8***
**Experiment 3** ILT	Enrolled	12	15	8	13
	No Lesion	0	3	2	2
	Haemorrhage	1	2	1	6
	Outlier	0	0	0	0
	***Included***	***11***	***10***	***5***	***5***

### Statistical analysis

Group size was determined based on a power calculation previously described in Ashioti et al. (2007). All statistical analyses were performed using the Statistical Package for Social Science (SPSS version 13). A GLM repeated measures ANOVA was performed on all MRI and bilateral asymmetry test data to detect overall differences between groups. This was followed by a one-way ANOVA to observe any differences at each individual time-point. A non-parametric Kruskall-Wallis test was performed on all neurological scoring data to detect differences between groups at each time-point. All MRI and bilateral asymmetry test and neurological scoring data are expressed as mean ± SEM. A P value of less than 0.05 was chosen as the significance level for all analyses.

### Experiment 1: Neuroprotection in an extensive distal MCAo lesion

#### Animals and Design

A 120 minutes distal MCA occlusion generated an extensive, but very consistent cortical lesion to probe neuroprotection [13]. As described above, SHR rats were occluded 120 minutes using the distal transient MCAo model to induce extensive damage to the cortex without affecting subcortical structures. This was measured 1 and 3 days following infarction by MRI. As previously reported, this model also induces a very consistent neurological deficit that is strongly correlated to the extent of damage [13]. To determine if neuroprotection can be achieved under these conditions, a separate cohort of animals received the NMDA antagonist MK-801 as a positive control.

### Experiment 2: Neuroprotection in a mild distal MCAo lesion

#### Animals and Design

As prolonged focal ischaemia can diminish penumbral areas of 'tissue at risk', assessment of efficacy in a milder lesion model is therefore also necessary to determine potential neuroprotective effects under these conditions. A mild distal MCAo lesion induced by 60 minutes transient cerebral ischaemia in the distal MCAo model provides these conditions [13]. Evaluation of structural damage on day 1 and 3 by MRI and daily neurological deficits afforded a reliable assessment of acute neuroprotective effects. To establish the degree of potential neuroprotection under these conditions, the reference compound MK-801 was included as a positive control in a separate cohort of animals.

### Experiment 3: Efficacy in a combined cortical and sub-cortical infarct

#### Animals and Design

To ensure that neuroprotective effects are not model specific, potential therapeutic efficacy requires assessment in a second model of focal ischaemia (STAIR, 1999). Additionally, a longer assessment frame can provide a more extensive investigation of potentially delayed neuroprotective effects. Animals therefore underwent MRI scanning on day 1, 7 and 14 with concomitant behavioural assessment (bilateral asymmetry test, see below) in addition to daily neurological scoring. The reference compound MK-801 was investigated as a positive control in a separate cohort of animals.

##### Bilateral Asymmetry Test (BAT)

For this, sticky tape (Dudley, UK) approximately 6 cm long and 0.5 cm wide was placed around both forepaws of the animal [14]. The animals were then placed within a holding cage and the latency in seconds to remove the strips of tape from both forepaws was recorded. Two trials per animal, alternating the order of tape application, i.e. right versus left, were recorded for each time point. Animals were assessed prior to MCAo surgery (-2 days) to provide a baseline measure and at 3 and 14 days post-treatment to evaluate the potential behavioural effects of neuroprotection. For statistical analyses, a GLM repeated measures ANOVA was calculated to establish a main group effect. This was followed by one-way ANOVAs to determine differences at each time-point.

## Results

### Experiment 1: Neuroprotection in a extensive distal MCAo lesion

**Figure 1:**
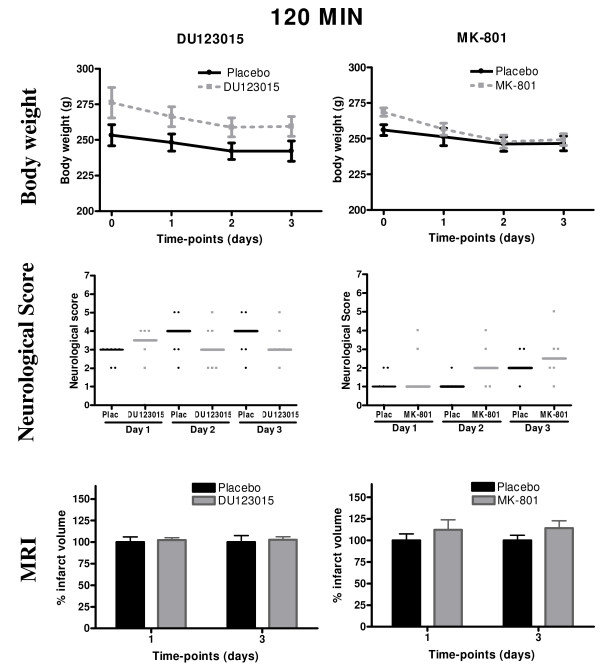
**Neuroprotective efficacy of 5HT1A agonist DU123015 and positive control MK-801 in the 120 minutes distal MCAo model**. The body weights (absolute weight in grams ± SEM) did not differ significantly for DU123015- (A) or MK-801-treated subjects (B). (C) No significant differences on neurological deficit were found for DU123015- (D) or MK-801-treated animals (E). No significant differences between groups were observed for oedema corrected MRI lesion volumes for DU123015-treated animals and glucosaline control treated animals (F). Also, no significant reduction in lesions was detected using the positive control compound MK-801.

As a general indication of recovery, post-operative weight was recorded daily. In both DU123015 and glucosaline control-treated groups, post-operative weight did not appear to alter greatly from the pre-operative weight (Figure 1A). This was also observed in the MK-801 treated and saline treated control groups (Figure 1B) indicating that neither compound exerted any deleterious effect on post-operative recovery. Neurological scoring assesses the impact of damage on neurological functions to provide a basic evaluation of functional impairments and potential minimisation of deficits by neuroprotection. DU123015 treated and control animals exhibited a mild neurological deficit (score of 3.5) that remained stable over the first 3 days post-surgery (Figure 1C). However, there was no significant difference between DU123015 and glucosaline-treated animals. Likewise, no significant difference between MK-801 treated and control animals was detected on the neurological scale (Figure 1D).

Differences in infarct volume were assessed using a GLM repeated measures ANOVA which indicated that DU123015 did not significantly impact on the overall volume of the 120 minutes ischaemic lesion compared to controls at either 1 or 3 days. The infarct volume of the DU123015 treated group increased by 8% ± 5 at day 1 and by 10% ± 4 at day 3 compared to controls. This indicates a minor exacerbation of the lesion due to the presence of the DU123015 compound. However, this was not statistically significant (F_(1,11) _= 1.505, P = 0.245) (Figure 1E). The use of MK-801 as a positive control in this study also did not decrease infarct volume compared to its controls. No significant difference in infarct volume between MK-801 treated and control animals was observed (F_(1,9) _= 1.206, P = 0.301) (Figure 1F).

### Experiment 2: Neuroprotection in a mild distal MCAo lesion

**Figure 2:**
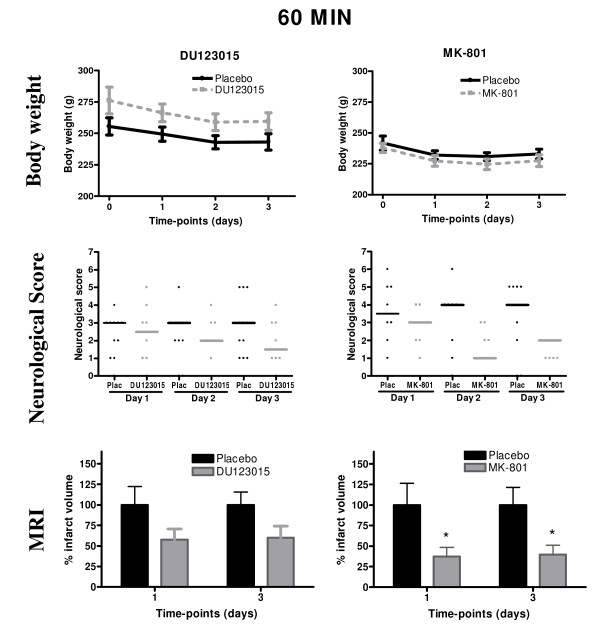
**Neuroprotective efficacy of the 5HT1A agonist DU123015 and positive control MK-801 in the 60 minutes distal MCAo model**. (A) The body weights of animals treated with DU123015 and glucosaline-treated controls. No significant differences between groups were observed. (B) The body weights for MK-801 and saline-treated controls. No significant differences between groups were observed. Neither DU123015 (C), nor MK-801 (D), significantly improve neurological functions, although on day 2 MK-801 almost significantly improved function (P = 0.056). (E) Oedema corrected MRI lesion volumes did not differ for DU123015-treated animals and glucosaline controls. (F) MK-801-treated animals exhibited a significant reduction in lesion volume at both time-points. (**) P < 0.01, (*) P < 0.05.

The body weight of both DU123015-treated and its control group was stable across time and did not change from baseline body weight (Figure 2A). Likewise, no detrimental impact of surgery or treatment was detected in the MK-801-treated group when compared with control animals (F_(1,15) _= 0.824, P = 0.378, Figure 2B). All animals exhibited neurological deficits. However, a Kruskal-Wallis non-parametric test revealed no significant difference between DU123015 and control treated animals (Figure 2C). The MK-801 treated animals performed better at neurological tasks than their respective controls at all time-points post-surgery. Although there is no significant difference in neurological deficits between MK-801 and control-treated animals, a Kruskal-Wallis test indicates a borderline significant improvement after MK-801 treatment on day 2 (P = 0.056) (Figure 2D).

Hyperintense regions were detected within the cortex in T_2_-weighted MRI images at both 1 and 3 days post MCAo. A GLM repeated measures ANOVA revealed no significant differences between the lesion volumes of the DU123015 treated animals and control animals (F_(1,17) _= 2.304, P = 0.147). A power calculation was also performed on this data and revealed that the number of animals required within each group to reach a statistical significant difference is 35. This number is too large for pre-clinical experiments and it was therefore deemed as unethical to reinvestigate DU123015 in this model (Figure 2E). However, in comparison MK-801 significantly reduces infarct size by 62% ± 12 1 day post surgery and 56% ± 11 3 days post-surgery (F_(1,15) _= 7.496, P < 0.05). A one-way ANOVA further revealed that the MK-801 treated group is significantly different from controls at both time-points post-occlusion (day 1 P < 0.05, day 3 P < 0.05, Figure 2F).

### Experiment 3: Effect of DU123015 on a combined cortical and sub-cortical infarct

**Figure 3:**
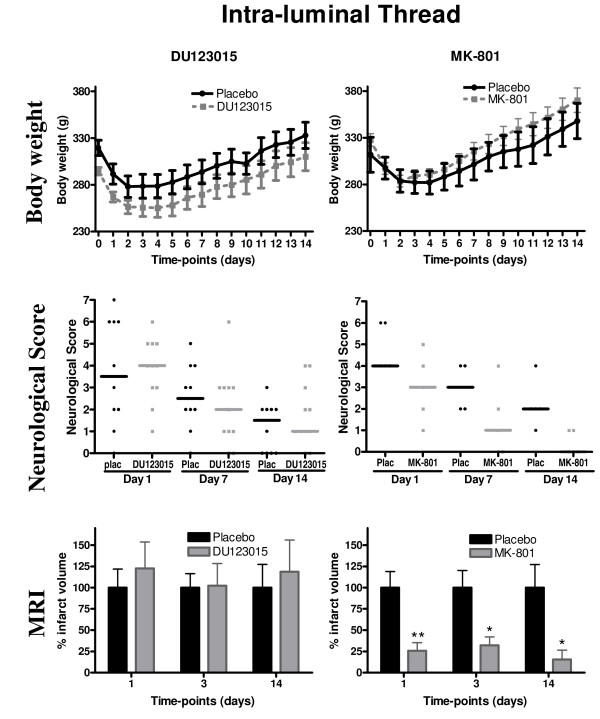
**Neuroprotective efficacy of 5HT1A agonist DU123015 and positive control MK-801 in the 90 minutes Intra-Luminal Tread (ILT) model of MCAo**. The body weights of animals treated with DU123015 (A) and MK-801 (B). (C) No significant differences were observed on neurological functions in animals treated with DU123015 (D) A Kruskall-Wallis non-parametric test indicated that MK-801 improved neurological functions impaired by stroke. (E) No significant differences were observed on oedema corrected MRI lesion volume for DU123015-treated animals. (F) In contrast, MK-801 significantly reduced lesion volume at all time points (*) P < 0.05 (**) P < 0.01.

The intraluminal thread model has a greater impact on the animals' general physiology and this is reflected in the greater weight loss and time to recover to pre-operative weight. The maximum weight loss for both the DU123015-treated animals and controls was observed on days 2 to 4 (Figure 3A). However, pre-operative weight recovered by 14 days post-surgery. An independent t test revealed a significant difference in pre-operative weight between DU123015 and placebo treated groups (P < 0.05). However, A GLM repeated measures ANOVA revealed no significant differences between the weights of DU123015-treated animals and their respective controls at all other time-points (F_(1,19) _= 2.069, n.s.). MK-801 showed an improved weight gain from 1 to 2 weeks post-surgery compared to controls, although this did not reach statistical significance (Figure 3B, F_(1,8) _= 0.566, P = 0.473). All animals recovered to pre-operative weight between day 9 and day 10. Treating animals with MK-801 or DU123015 therefore did not significantly accelerate recovery of pre-operative weights.

DU123015 did also not significantly improve the animals' neurological deficits at any time-point post-surgery. The DU123015-treated group recovered from a neurological score of 4 to 1 from day 1 to day 14, but this improvement was akin to placebo-treated controls and did not reach statistical significance. MK-801-treated animals had a consistently improved neurological outcome at all time-points (P < 0.05). MK-801 therefore had a beneficial effect on neurological recovery, whereas DU123015 did not significantly alter this outcome.

The T_2_-weighted MRI scans at 1, 3 and 14 days post MCAo did also not reveal any protection of brain tissue after administration of DU123015 (Figure 3E, F_(1,19) _= 0.078, n.s.), but rather indicated a slight increase in infarct volume by 16% ± 26 1 day post-surgery and by 11% ± 32 at 14 days relative to placebo-controls. Cortical only and striatal only infarct volumes were also assessed for both DU123015 and placebo-controls. However, this analysis also revealed no significant differences between cortical only (F_(1,19) _= 0.076, P = 0.786) and striatal only (F_(1,19) _= 0.317, P = 0.580) lesion volumes of DU123015 treated animals when compared to controls. In contrast, MK-801 consistently decreased lesion volume at all time points (Figure 3F, F_(1,8) _= 10.179, P < 0.05). This decrease in lesion volume was substantial and resulted in a 67% ± 11 decrease in lesion volume on day 1, 66% ± 8 reduction on day 3 and a final reduction of 80% ± 13 at day 14 relative to placebo-controls. This demonstrates that the therapeutic effect of MK-801 is persisting and increasing over at least 2 weeks. MK-801 therefore is efficacious in this model, whereas DU123015 does not convey neuroprotection.

**Figure 4:**
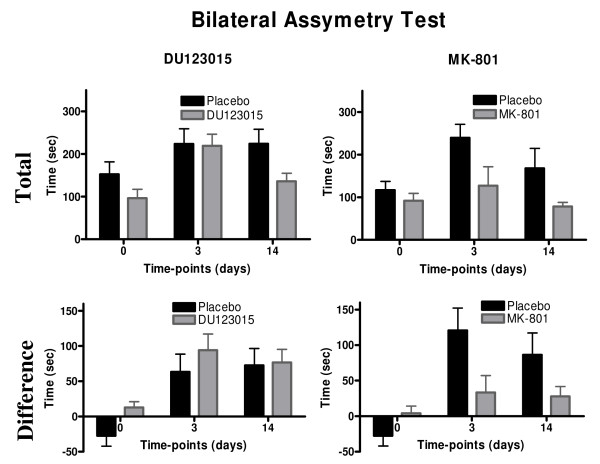
**Bilateral Asymmetry Test (BAT) for 90 minutes intra-luminal thread model of MCAo**. DU123015 did not improve the total time taken to remove sticky tape from forepaws (A), whereas MK-801 did significantly improved outcome (B) Difference between removal of the sticky tape from the right and left forepaws (L-R) did not improve with DU123015 treatment (C), whereas MK-801 did (D).

To further probe the behavioural significance of a potential neuroprotectant, the impact of treatment on somatosensory function was assessed using the bilateral asymmetry test (BAT). A GLM repeated measures ANOVA revealed no overall statistically significant differences in total time taking for tape removal between DU123015 and glucosaline control-treated groups (Figure 4A, F_(1,19)_= 1.518, n.s.). A similar observation was evident on the difference between removing the left or right sticky tape (a measure of sensorimotor neglect), where DU123015-treated and control animals performed alike (Figure 4C, F_(1,14)_= 0.471). In contrast, MK-801 significantly improved the animals' behaviour and reduced removal time by 54% at 14 days. A GLM repeated measures ANOVA revealed a main effect of group at both 3 and 14 days post-surgery (Figure 4B, F_(1,8) _= 8.724, P < 0.05). An even more dramatic effect was evident on the forepaw asymmetry that was reduced by 73% on day 3 and by 69% on day 14 (Figure 4D, F_(1,8) _= 9.214, P < 0.05). Therefore MK-801 not only exerted an anatomical benefit, but demonstrated a corresponding functional improvement.

## Discussion

Pharmacological therapies for stroke aim to provide neuroprotection that results in an improved behavioural outcome. 5HT_1A _agonists have recently emerged as a potential new target to promote neuroprotection in stroke. However, to date very few thorough preclinical examinations using this class of compounds have been conducted and we therefore undertook here a systematic study of the novel 5HT_1A _agonist, DU123015. Using a variety of transient focal cerebral ischaemia models that replicate the pathophysiology of stroke, no significant therapeutic efficacy of DU123015 was detected on structural and behavioural measures. In contrast, MK-801, an NMDA-antagonist, which served as a positive control significantly reduced lesion volume and behavioural impairments in 2 of the 3 experiments.

### Efficacy of 5HT1A agonists

Oedema corrected MR images, neurological deficit scoring and the Bilateral Asymmetry Test (BAT), all revealed that DU123015 did not significantly improve outcome in a mild or severe cortical infarct. Also, the location of the lesion itself did not affect efficacy, as neither a purely cortical, nor a cortical plus subcortical lesion, bestowed any efficacy to the 5HT_1A _agonist. In contrast, MK-801 exerted significant benefits in all cases, apart from the 120 min cortical lesion suggesting that this occlusion time is too severe to observe therapeutic efficacy. The use of hypertensive or normotensive animals also did not affect the structural or behavioural outcome with DU123015 proving that no indirect benefit can be gained through an anti-hypertensive effect. Increasing the dose of DU123015 could potentially yield neuroprotection, but physiological effects of higher doses on temperature and blood pressure could be the main mediators of lesion attenuation at these doses. Previous studies using similar doses as those reported here have indeed observed a therapeutic benefit of 5HT_1A _agonists in cell culture [8,19], models of traumatic brain injury [17], and permanent occlusion models of ischaemia [9,20-23].

To ensure a very thorough assessment of treatment efficacy, we used here a multi-modal assessment that included structural and behavioural measures that are interdependent [13]. To date, only a few studies [11,12] have reported significant treatment effects 5HT_1A _agonists in stroke, but these mainly relied on a single outcome measure. In contrast, the present series of experiments provides a strong indication that the 5HT_1A _agonist DU123015 at this dose does not promote significant structural, neurological or behavioural improvements in stroke.

Although the pharmacological characteristics of DU123015 are comparable to Repinotan, the recent failure of Repinotan in a phase II, randomized, double blind clinical stroke study has provided some uncertainty as to the reliability and predictability of some of these earlier pre-clinical studies [24]. No improvement in behavioural or neurological outcomes was evident when patients were treated with Repinotan compared to placebo. Our results here are in line with these clinical observations that our 5HT_1A _agonist did not affect the anatomical, behavioural and neurological outcomes after stroke.

### Assessing neuroprotection in stroke

The failure of over 700 potential neuroprotective compounds which have been successful in pre-clinical studies has prompted the development of more rigorous guidelines for future pre-clinical and clinical stroke trials. However, the recent failure of the most promising compound for the treatment of ischaemic stroke, NXY-059 [25], a free radical scavenging agent, has led to the stipulation that more stringent conceptual and methodological issues must be adopted in order to produce successful neuroprotective compounds [26]. For example, testing in two models of focal ischaemia (varying durations of ischaemia) in different strains of rats should be reported using stringent inclusion/exclusion criteria and outcome measures assessed at acute and chronic time-points using objective in-vivo approaches. This method of evaluating potential neuroprotective compounds will minimise the translation of less efficacious compounds and provide a more thorough pre-clinical evaluation of putative therapeutic agents

Furthermore, the inclusion of a positive reference compound validates the models, experimental methods and outcome measures each time [27]. We here utilized Dizolcilpine (MK-801) as a positive control due to extensively documented therapeutic effects of this compound in experimental stroke [18,28-30] and other studies previously also included this compound as a positive control [28,31,32]. Inclusion of the positive reference compound and its lack of efficacy in a severe cortical lesion here therefore allowed us to establish that any neuroprotection that could be seen under these conditions would indeed have been very significant, while at the same time providing us with evidence that a standard neuroprotection was achievable under the other conditions.

Establishing efficacy, however, is also dependent on stringent outcome measures. The interdependence of different measures is an important aspect to corroborate outcome measures within a single experiment. We previously reported a highly significant correlation of MRI lesion volume with neurological score and the BAT in the cortical infarct model but there was a less significant correlation between neurological score and the BAT [13]. The MRI outcome measure in this study was also the most consistent measure in terms of variability and hence provided a more reliable measure to detect group differences in the distal MCAo model. Notably, MK-801 decreased neurological deficit after mild ischaemic damage, but the high variability of neurological deficit in the control group limited our ability to detect a significant group difference, although a clear statistical difference could be detected from the MRI. In the ILT model, however, MK-801 significantly reduced both the neurological deficits and lesion volume demonstrating the reliability and sensitivity of both measures to detect a significant treatment effect. In contrast, the 5HT_1A _agonist lacked efficacy on any test, clearly indicating a lack of significant efficacy. The inherent variability in neurological tests may render this assessment modality less sensitive to neuroprotection compared to MRI, but neurological deficits, as well as behavioural tests measure aspects of the damage that are not completely predicted by structural measures. Nevertheless, these functional facets need to be included in preclinical studies to highlight primary clinical outcome measures that might not be entirely captured by anatomical assessments.

## Conclusion

This study has revealed that the 5HT_1A _agonist DU123015 was not efficacious on any outcome measure that was examined in this investigation. Our study also suggests a more rigorous approach to neuroprotection studies, which provides detailed evidence of the lack of neuroprotective efficacy of DU123015 in many different circumstances. We believe the publication of neuroprotection studies producing negative or neutral results is a vital aspect of screening novel compounds and drug targets. These are a necessary addition to the literature to establish the clinical potential of a class of agents [33]. Future guidelines for the screening of potential neuroprotective compounds and avoidance of false positives may benefit from further methodological advances as exemplified in this study.

## Authors' contributions

MA carried out all imaging, behaviour, neurological scoring, ILT MCAo surgery and EEG. She also performed all analysis and statistics and drafted the manuscript. JB performed all distal MCAo surgery, aided in the design and coordination and helped to draft the manuscript. AL provided the MRI sequences, analysis methods, aided in the design of the experiments and drafting the manuscript. MB aided in the design, analysis and interpretation of the EEG data. AM conceived of the study and aided in its design and coordination. MM aided in the design and coordination of the study, in the drafting of the manuscript and the analysis of the results. SW conceived the study, aided in its design and coordination and the drafting of the manuscript. All authors have read and approved the final manuscript.

## Supplementary Material

Additional file 1 **Effects of DU123015 on total cortical brain activity and physiological parameters when administered at 35.2 μg/kg i.v**. DU123015 significantly reduced global cortical brain activity by approximately 20% up to 1 hour post injection (A). However, no effect on both Blood Pressure (B) and O_2 _saturation (C) was observed following compound administration. Click here for file

Additional fle 2 **T_2_-weighted MRI images**. MR images of a central slice from a representative animal in all MCAo groups observed with and without the intervention of DU123015 and MK-801 at day 1. Click here for file
