# Supplementary figures and images for: Neither in vivo MRI nor behavioural assessment indicate therapeutic efficacy for a novel 5HT1A agonist in rat models of ischaemic stroke
Source: BMC Neurosci. 2009 Jul 16;10:82. doi: 10.1186/1471-2202-10-82 (PMC2720976; doi:10.1186/1471-2202-10-82)

A

## Total Energy

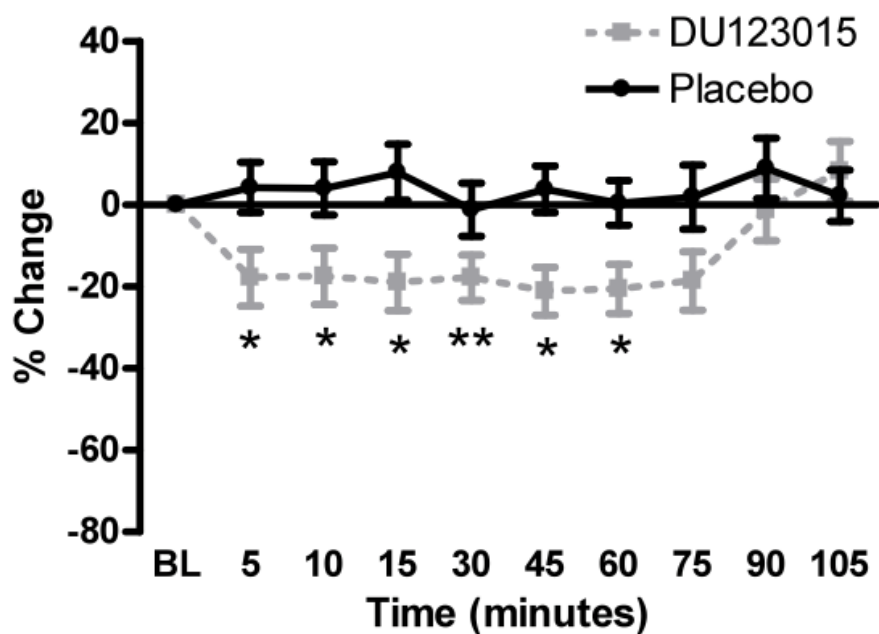

B

## Blood Pressure

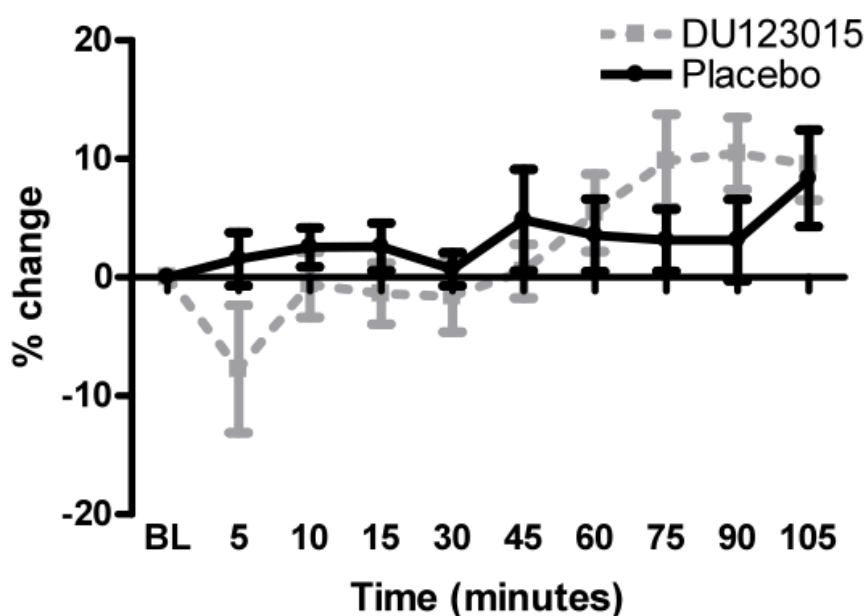

C

O<sub>2</sub> saturation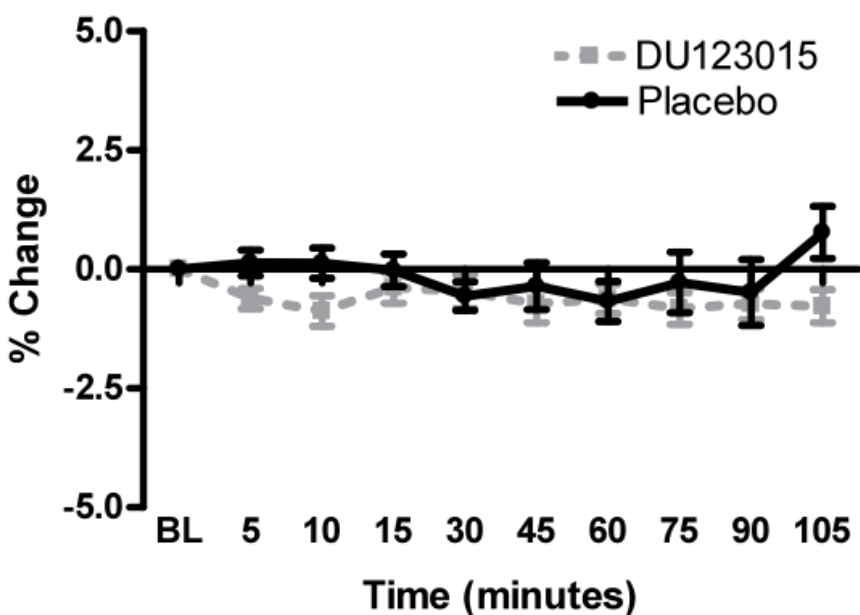

Supplement: Additional file 1 — Effects of DU123015 on total cortical brain activity and physiological parameters when administered at 35.2 μg/kg i.v. DU123015 significantly reduced global cortical brain activity by approximately 20% up to 1 hour post injection (A). However, no effect on both Blood Pressure (B) and O2 saturation (C) was observed following compound administration. [file 1471-2202-10-82-S1.pdf]

DU123015

Placebo

MK-801

Placebo

120

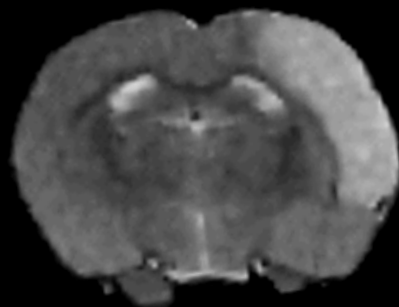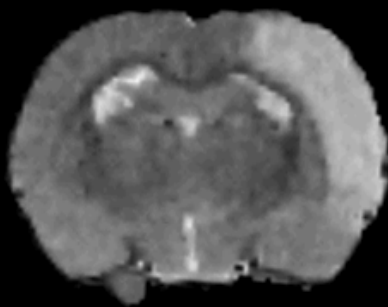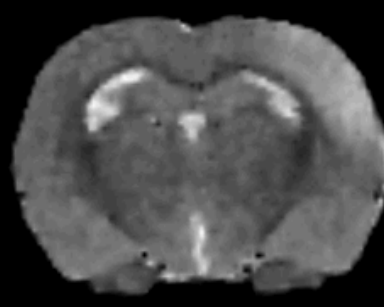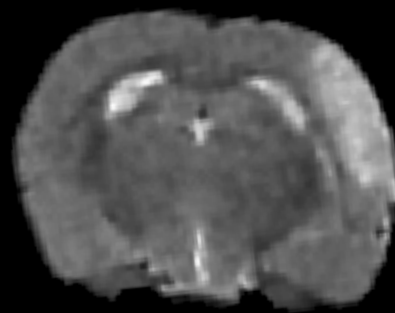

60

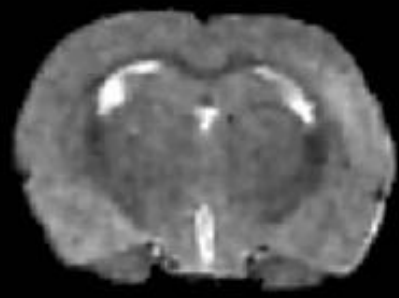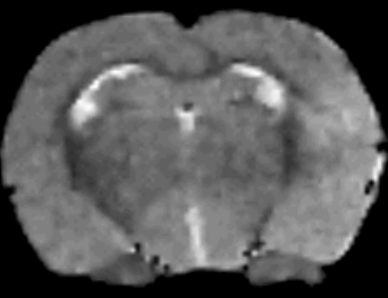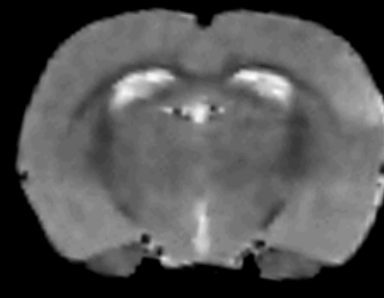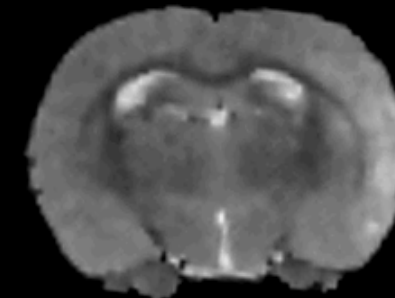

ILT

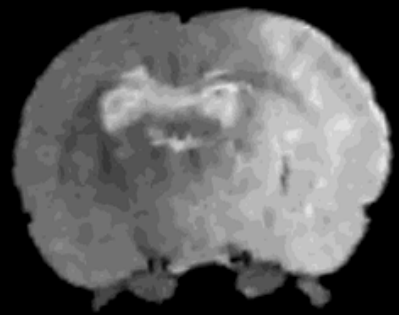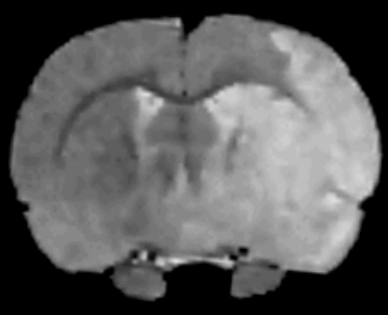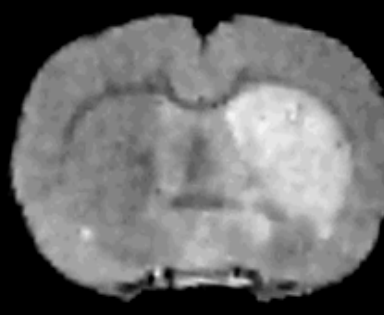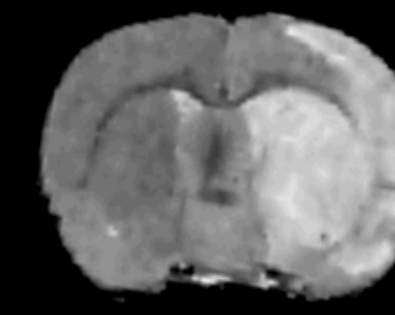

Supplement: Additional fle 2 — T2-weighted MRI images. MR images of a central slice from a representative animal in all MCAo groups observed with and without the intervention of DU123015 and MK-801 at day 1. [file 1471-2202-10-82-S2.pdf]
